# How to Evolve a Perianth: A Review of Cadastral Mechanisms for Perianth Identity

**DOI:** 10.3389/fpls.2018.01573

**Published:** 2018-10-29

**Authors:** Marie Monniaux, Michiel Vandenbussche

**Affiliations:** Laboratoire Reproduction et Développement des Plantes, Université de Lyon, ENS de Lyon, UCB Lyon 1, CNRS, INRA, Lyon, France

**Keywords:** perianth, flower, evolution, petal, sepal, ABC model

## Abstract

The flower of angiosperms is considered to be a major evolutionary innovation that impacted the whole biome. In particular, two properties of the flower are classically linked to its ecological success: bisexuality and a differentiated perianth with sepals and petals. Although the molecular basis for floral organ identity is well understood in extant species and summarized in the famous ABC model, how perianth identity appeared during evolution is still unknown. Here we propose that cadastral mechanisms that maintain reproductive organ identities to the center of the flower could have supported perianth evolution. In particular, repressing B- and C-class genes expression toward the inner whorls of the flower, is a key process to isolate domains with sepal and petal identity in the outer whorls. We review from the literature in model species the diverse regulators that repress B- and C-class genes expression to the center of the flower. This review highlights the existence of both unique and conserved repressors between species, and possible candidates to investigate further in order to shed light on perianth evolution.

## Introduction

Flowering plants (angiosperms) gather more than 350,000 species, a stunning number in regard to all other land plants that count no more than 35,000 species (The Plant List, 2013). This dominance of angiosperms might be partly due to the flower, a highly efficient structure for reproduction (Regal, 1977). The flower has some key features such as bisexuality, a closed carpel, and a perianth (i.e., the structure that surrounds the reproductive organs, typically organized in sepals, and petals) that can attract pollinators and therefore participate in the speciation process (Fenster et al., 2004). This is mainly supported by the petals, that display a complex set of traits such as color, fragrance, shape, or epidermal cell patterns (Glover, 2014). Petals can also assist in flower opening (van Doorn and Van Meeteren, 2003), while sepals mainly protect the other floral organs. In this review we will use the term petal and sepal as a functional definition for all petaloid (showy and playing an attractive role) and sepaloid (greenish and playing a protective role) organs, respectively, irrespective of their position in the flower. With this definition, all petals (and all sepals) are therefore not necessarily homologous organs (Ronse De Craene and Brockington, 2013).

Although recent research has led to considerable progress on the question of the origin of the flower (Moyroud et al., 2017; Sauquet et al., 2017), large questions are still open. In particular, the timing and order of events leading from the reproductive structure of the most recent common ancestor of seed plants – likely a unisexual structure without perianth – to the ancestral flower – likely a bisexual flower with an undifferentiated perianth of petals – is still unknown (Sauquet and Magallón, 2018). These events include transition from unisexuality to bisexuality, compression of the reproductive axis, evolution of a perianth and evolution of a closed carpel (Specht and Bartlett, 2009; Sauquet and Magallón, 2018; Scutt, 2018). Despite this uncertainty it seems reasonable to assume that the perianth evolved last, after bisexuality, axis compression and carpel evolution (Baum and Hileman, 2007). Later, the perianth often differentiated into an outer whorl of sepals and an inner whorl of petals (resulting in a so-called differentiated or bipartite perianth), which is particularly representative of core eudicots (Specht and Bartlett, 2009).

The origin of the perianth is still unresolved, but anatomical and developmental observations can shed some light on it. Sepals from most angiosperms have a leaf-like appearance suggesting they have a direct bract or leaf origin. Petals likely arose multiple times during evolution (Kramer and Irish, 2000) with two possible origins: bracteopetals that evolved from bracts and andropetals that evolved from stamens. Bracteopetals are typically observed in basal angiosperms that show a continuous differentiation between bracts and petaloid organs (Ronse De Craene, 2007). In contrast andropetals appear restricted to a few clades (Ranunculales and Caryophyllales for instance) where petals have probably been lost and reinvented (Ronse De Craene, 2007; Brockington et al., 2012; Ronse De Craene and Brockington, 2013). However, for most angiosperm species, the origin of petals remains unclear and a combination of anatomical and genetic work are needed to discriminate between the two possibilities.

Genetic work on model species have provided molecular support for the key events accompanying flowering: formation of the flower meristem, specification of floral organ identities (the famous ABC model), floral organ outgrowth and maturation, and fertilization (Glover, 2014). Based on this data, molecular models for the evolution of floral structures such as the bisexual axis have been proposed (Baum and Hileman, 2007; Frohlich and Chase, 2007; Specht and Bartlett, 2009). A similar approach can be followed to generate molecular hypotheses for the origin of the perianth; more specifically to speculate how an identity domain for the perianth could have emerged from an ancestral flower containing only reproductive organs. Here we propose that cadastral mechanisms maintaining reproductive identity to the center of the flower could have supported perianth appearance during evolution. Based on genetic work in model species, we review some of the molecular players underlying these cadastral mechanisms.

## Creating a Domain for Perianth Identity

**FIGURE 1:**
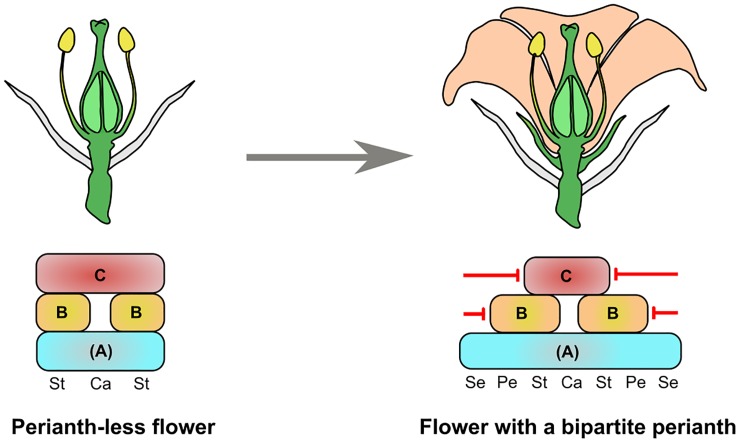
Model for the origin of a bipartite perianth from a perianth-less (ancestral) flower. The ancestral flower is composed of bracts (gray organs), stamens (St), and carpels (Ca). The flower with a bipartite perianth has bracts, sepals (Se, green organs), petals (Pe, orange organs), stamens, and carpels. The identity of all these organs is specified by an (A)BC model, and the restriction of the B- and C- gene classes to the center of the flower is a key process for perianth identity to be specified. One possibility for this is the specific repression of B- and C-class genes (red arrows) to their respective expression domains.

Assuming that the perianth was the last angiosperm synapomorphy to appear, we have asked the following question (Figure 1): how could a typical flower with 4 organ identities have been generated from a bisexual perianth-less flower with 2 organ identities?

In Figure 1, we use a genetic framework based on the (A)BC or FBC models proposed by Causier et al. or Baum and Hileman, respectively, where (A) or F is a floral identity function acquired at early stages of floral meristem development and necessary for all floral organ identities (Baum and Hileman, 2007; Causier et al., 2010). We chose not to use the classical ABC model because the existence of the A function is much debated (Schwarz-Sommer et al., 1990; Litt, 2007; Causier et al., 2010; Heijmans et al., 2012; Morel et al., 2017). With the (A)BC framework, floral organ identities from a typical angiosperm flower are specified by a combination of (A) expression for sepals, (A) + B for petals, (A) + B + C for stamens and (A) + C for carpels. Similarly, we can assume that the perianth-less ancestral flower had floral organ identities specified by (A) + B + C for stamens and (A) + C for carpels. In support of this, the B- and C-class functions are remarkably conserved across angiosperms, and gymnosperm male and female cones also show B + C and C gene expression, respectively (Sundström and Engström, 2002; Becker and Theissen, 2003; Zhang et al., 2004; Moyroud et al., 2017).

**FIGURE 2:**
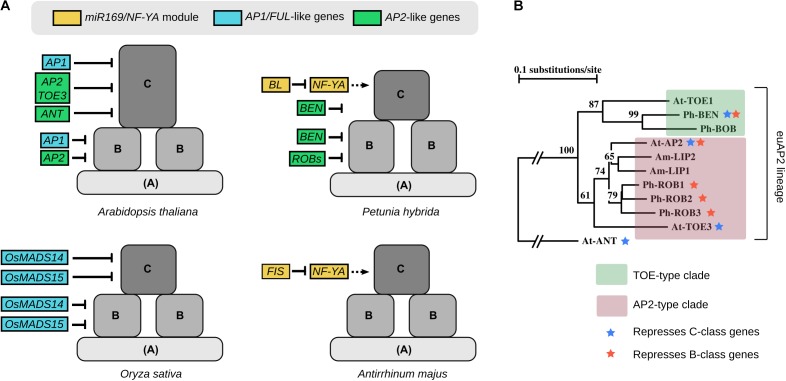
**(A)** Summary of some of the regulators involved in B- and C-class gene repression in Arabidopsis, Petunia, Rice, and Antirrhinum. The color code indicates their membership to modules or gene families that were found to be recurrent between species. Dotted arrows for NF-YA indicate that their hypothetical role in C-gene activation has not been demonstrated so far. **(B)** Simplified phylogeny of AP2-like proteins, showing the euAP2 lineage composed of the TOE-type and AP2-type clades. Blue and orange stars indicate when the proteins were shown to repress C-class and B-class genes expression, respectively. Simplified from (Morel et al., 2017). At, *Arabidopsis thaliana*; Ph, *Petunia hybrida*; Am, *Antirrhinum majus*.

Therefore, the difference in gene expression between the perianth-less and bipartite perianth flower mainly resides in the peripheral expression domains of the B and C genes. This suggests that cadastral mechanisms that maintain (through repression or activation) B- and C-class gene expression to their dedicated area could have been key for perianth evolution. From reviewing the literature, it appears that several repressors of B- and C-class gene expression have been identified in extant species. Indeed, specifically repressing B genes from the first whorl and C genes from the first and second whorls is one possible way to generate a domain sufficient for sepal and petal identity to be specified. In the following paragraphs we will review the repressors of B- and C-class genes and examine how conserved their function is across angiosperms (Figure 2A). This review is mostly based on functional studies in the model species *Arabidopsis thaliana* (Arabidopsis), *Petunia hybrida* (Petunia), *Oryza sativa* (Rice), and *Antirrhinum majus* (Antirrhinum).

## Repressors of C-Class Gene Expression

A-class genes from the classical ABC model were proposed, already from the beginning, to have a dual function in specifying organ identity in the first two whorls (sepals and petals, alone or in combination with B-genes), and repressing the C function from these same whorls (Coen and Meyerowitz, 1991). In Arabidopsis, *APETALA1* (*AP1*) and *AP2* are generally classified as A-class genes, although *AP1* was added only later to this class (Bowman et al., 1991; Coen and Meyerowitz, 1991; Gustafson-Brown et al., 1994). Indeed, AP1 does repress C-class gene expression from young flowers, by forming complexes with the flower meristem identity regulators SHORT VEGETATIVE PHASE (SVP) or AGAMOUS-LIKE24 (AGL24) and the general floral repressors LEUNIG (LUG) and SEUSS (SEU) (Gregis et al., 2006, 2009; Sridhar et al., 2006). But examination of *ap1* in combination with other mutations revealed that *AP1* is not strictly necessary for petal and sepal identity, since these organs can develop in some *ap1* mutant backgrounds (Yu et al., 2004; Causier et al., 2010). Moreover outside of Brassicaceae, *AP1* orthologues are generally not needed for sepal and petal identity (Litt, 2007; Causier et al., 2010). For instance in Antirrhinum, mutant in the *AP1* ortholog *SQUAMOSA* shows defects in floral meristem identity but not necessarily in perianth identity (Huijser et al., 1992). In contrast in Rice, mutations in the *AP1*/*FRUITFUL (FUL)* lineage members *OsMADS14* and *OsMADS15* results in the extension of C-class gene expression (and to a lesser extent, B-class gene expression) in the outer whorls of the flower, therefore leading to palea-to-carpel and lodicule-to-stamen homeotic conversions (Wu et al., 2017).

In Arabidopsis the other A-class gene *AP2* represses *AGAMOUS (AG*, the C-class gene*)* expression in whorls 1 and 2, and the *ap2* mutant shows the sepal-to-carpel and petal-to-stamen homeotic conversions expected from an A-class mutant (Drews et al., 1991) (see Figure 2B for a simplified phylogeny of *AP2*-like genes). Both the *AP2*-type gene *TARGET OF EAT 3* (*TOE3*) and the *AP2*-like gene *AINTEGUMENTA* (*ANT*) also repress *AG* expression; however, homeotic changes in the corresponding mutants are very subtle, if any (Krizek et al., 2000; Jung et al., 2014). *AP2* and *TOE3* expression is regulated at the translational level by the microRNA *miR172* (Chen, 2004; Wollmann et al., 2010). In Petunia, it was recently found that the *euAP2* clade member *BLIND ENHANCER* (*BEN*), although not the ortholog of *AP2*, also represses C-class gene expression from the first whorl (Morel et al., 2017); in maize mutations in the *AP2*-like genes *ids1* and *sid1* result in ectopic expression of the *AG*-like genes *zag1* and *zmm2* in bracts that become carpelloid (Chuck et al., 2008); in other species antagonistic expression patterns suggest a similar repressive role of *AP2*-like genes on C-class gene expression (Yang et al., 2015). In contrast, the Antirrhinum *AP2* orthologs *LIPLESS1* (*LIP1*) and *LIP2*, and the petunia *AP2* orthologs *REPRESSOR OF B-FUNCTION 1 (ROB1), ROB2*, and *ROB3* play a role in sepal and petal development but do not seem to antagonize C-class gene expression in the perianth (Keck et al., 2003; Morel et al., 2017). Overall, this shows that members of the *euAP2* lineage are often involved in C-class gene repression in the outer whorls of the flower, but this role sometimes has been swapped between members of the lineage, switching between *AP2*-type and *TOE*-type clade members (Figure 2B). These two clades predate the monocot-eudicot divergence (Kim et al., 2006), suggesting that the repression of C-class genes by members of the *euAP2* lineage might be relatively ancient.

In Arabidopsis, other C-class repressors have been identified: the SUPERMAN-like zinc finger protein RABBIT EARS (RBE) represses *AG* in whorl 2 (Krizek et al., 2006), while the bZIP transcription factor PERIANTHIA (PAN), together with SEU, represses *AG* in whorl 1 (Das et al., 2009; Wynn et al., 2014). However, mutations in these genes are not sufficient to cause homeotic changes in floral organs, showing that a certain threshold of *AG* ectopic expression is needed for homeotic conversion. In contrast, completely different C-class repressor genes have been identified in Petunia and Antirrhinum. Indeed in both species a member of the *miR169* family, *BLIND* (*BL*) in Petunia and *FISTULATA* (*FIS*) in Antirrhinum, represses C-class gene expression in the outer whorls, possibly by targeting members of the NF-YA family for degradation (Cartolano et al., 2007). Members of the miR169/NF-YA module from various species have been involved in several developmental processes such as flowering time, root architecture, embryogenesis, general responses to stress or interaction with pathogens (Laloum et al., 2013; Zhao et al., 2016; Zanetti et al., 2017) but apart from Petunia and Antirrhinum, never in floral patterning. This specific function of miR169/NF-YA might thus have evolved only in the euasterids lineage, unless in rosids it is hidden by redundancy between the multiple copies of *miR169* and *NF-YA* genes.

Other C-class repressors with a broader spatial action have been found, including BELLRINGER that represses *AG* expression in the Arabidopsis stem, inflorescence meristem and young flower meristem (Bao et al., 2004); CURLY LEAF that represses *AG* expression in all vegetative tissues of Arabidopsis and Brachypodium (Goodrich et al., 1997; Lomax et al., 2018); STERILE APETALA that represses *AG* expression in the outer floral whorls and in the inflorescence meristem of Arabidopsis (Byzova et al., 1999); and FILAMENTOUS FLOWER that represses *AG* expression in the outer floral whorls and in the peduncle of Arabidopsis (Chen et al., 1999). Mutations in each of these genes cause homeotic defects in Arabidopsis floral organs. The fact that these repressors are not spatially specific to the outer floral whorls does not exclude that they could have played a role in perianth evolution by being coopted for repression of *AG* in whorls 1 and whorls 2, while they already repressed *AG* expression from other tissues (True and Carroll, 2002). However, since their role has hardly been investigated in other species than Arabidopsis so far, we do not know how conserved these mechanisms are and if they could have been involved in perianth evolution.

## Repressors of B-Class Gene Expression

In contrast to the many C-class genes repressor identified, fewer genes have been shown to repress B-class gene expression from the first whorl of the flower (Figure 2). In Arabidopsis, AP2 represses the B-class genes *APETALA3* (*AP3*) and *PISTILLATA* (*PI*) expression in whorl 1. While this is not evident from the phenotype of single *ap2* mutants, this becomes visible when an *ap2* mutant allele is present in an heterozygous state in the *topless* (*tpl*) mutant background, resulting in a partial sepal-to-petal conversion due to ectopic *PI* and *AP3* expression in whorl 1 (Krogan et al., 2012). But by far, the clearest evidence of B-class gene derepression in whorl 1 is found in Petunia in the quadruple *ben rob1 rob2 rob3* mutant, that shows an almost perfect homeotic conversion of sepals into petals (Morel et al., 2017). As such this flower is reminiscent of the undifferentiated perianth found in many angiosperm species, such as tulip or magnolia, and likely a characteristic trait of the ancestral flower (Sauquet et al., 2017). As previously mentioned, *BEN* and *ROBs* are all members of the *euAP2* lineage (Figure 2B). The single *ben* mutant also shows some petaloid sectors in sepals, indicating that BEN and ROBs partially redundantly repress B-genes expression in whorl 1 (Morel et al., 2017). Therefore, repression of B-genes expression by *euAP2* genes appears conserved between Arabidopsis and Petunia, suggesting that this regulation might have originated prior to the rosids-asterids divergence.

*APETALA1* has also been proposed to repress *PI* and *AP3* expression in Arabidopsis, when part of the repressive complex with AGL24, SVP, LUG, and SEU (Gregis et al., 2006, 2009), but whether AP1 is directly involved in this regulation is unclear. In Rice the *osmads14 osmads15* double mutant shows some derepression of B-class gene expression in whorl 1 but this ectopic expression does not seem strong enough to alter organ identity (Wu et al., 2017). Altogether, this suggests a somehow conserved role of *AP1/FUL* genes in repressing B-class gene expression but the evidence is scarcer than for *AP2* genes.

## Conserved and Unique Repressors of B- and C-Class Genes

Our review highlights that members of the large *AP2* family, and in particular the *euAP2* lineage, might have a conserved function in repressing both B- and C-class gene expression from the outer whorls of the flower, predating the rosids-asterids divergence. Hence members from this family are possible candidates to have played a role in perianth evolution. However, if *euAP2* genes were involved in both sepal and petal evolution, it would require uncoupling of their repressive action on B- and C-class genes, since B-class genes should be repressed in the first whorl only while C-class genes should be repressed in the first two whorls. In Arabidopsis, it is unknown how AP2 can have whorl-specific repressive action on B- and C-class genes, but it possibly resides in the interaction with different protein partners between whorls. In Petunia, euAP2 proteins repress B- and C-gene expression in the first whorl only, while repression of C-class genes in the second whorl is completed by *BL*, showing how the dual repressive function has been distributed between two sets of genes in this species. More functional studies in angiosperms, and particularly in early diverging taxa, are now needed to evaluate the possibility that *euAP2* genes were involved in perianth evolution.

Our review also shows that a large variety of repressors of C- (and to a more minor extent, B-) class genes exist in extant species. One might wonder in particular why so many C-genes repressors are found. None of the identified repressors are redundant with each other since single mutants in question all show ectopic C expression. Hence instead of conferring robust repression of C-class genes expression, these numerous repressors might actually provide a multiplicity of ways for evolution to relieve C-expression in the perianth. Since the perianth is a highly evolvable structure, i.e., a flexible trait that evolved multiple times during angiosperm evolution (Baum and Whitlock, 1999; Kramer and Irish, 2000; Hileman and Irish, 2009; Geuten et al., 2011), an hypothesis is that evolution could have tinkered with these various repressors for the perianth to appear or disappear in different taxa.

Petaloid features are not exclusively found on second whorl petals but often have been transferred to sepals, bracts or even stamens. Flower morphology is remarkably flexible and while true petals may have been lost, petaloidy shifted to analogous organs that acquired the petal traits needed for recognition by pollinators. These petaloid features are sometimes correlated with ectopic B-gene expression, like in many non-grass monocots for instance (Kanno et al., 2007), but there are also many cases of petaloid structures that have little or no B-genes expression, as well as non-petaloid structures that do express B-class genes, as reviewed in (Ronse De Craene and Brockington, 2013). We can argue that not all B-class genes might have been identified in species without a sequenced genome. Still, the evolution of petaloidy appears to be more complex than mere shifts in B-class gene expression, and the genes underlying these transfers of function from one organ to another might still remain to be identified.

## Conclusion

In this review we proposed that repressing reproductive organ identity to the center of the flower is a possible way for perianth identity to have emerged in the periphery of the flower, and we reviewed the B- and C-class genes repressors that have been identified in model species. Whether these repressors were actually evolved in perianth evolution some 150 million years ago remains of course hypothetical, and functional experiments in early-diverging gymnosperms and angiosperms are needed to evaluate such hypotheses. By far the largest source of variation in perianth morphology in angiosperms does not lie in flower patterning, but in changes in shape, color, scent or size of the petals, as beautifully illustrated in (Byng et al., 2018). The genetic basis for these variations, some of quantitative nature like spur length in *Aquilegia* (Yant et al., 2015), others of qualitative nature like presence or absence of pigmentation in Petunia (Hoballah et al., 2007), has only been identified on few occasions (Moyroud and Glover, 2017). These genes are likely downstream targets of B-class regulators, but how these master developmental genes direct the establishment of all petaloid features in a simultaneous manner, and which parts of this large network have been modified during evolution to generate morphological diversity, remains a mystery as big as perianth evolution itself.

## Author Contributions

All authors listed have made a substantial, direct and intellectual contribution to the work, and approved it for publication.

## Conflict of Interest Statement

The authors declare that the research was conducted in the absence of any commercial or financial relationships that could be construed as a potential conflict of interest.
